# CSF Presenilin-1 complexes are increased in Alzheimer’s disease

**DOI:** 10.1186/2051-5960-1-46

**Published:** 2013-08-06

**Authors:** María-Salud García-Ayllón, María-Letizia Campanari, Gunnar Brinkmalm, Alberto Rábano, Jordi Alom, Carlos A Saura, Niels Andreasen, Kaj Blennow, Javier Sáez-Valero

**Affiliations:** 1 Instituto de Neurociencias de Alicante, Universidad Miguel Hernández-CSIC, Av. Ramón y Cajal s/n, Sant Joan d’Alacant E-03550, Spain; 2 Centro de Investigación Biomédica en Red sobre Enfermedades Neurodegenerativas (CIBERNED), Barcelona, Spain; 3 Unidad de Investigación, Hospital General Universitario de Elche, FISABIO, 03203 Elche, Spain; 4 Department of Psychiatry and Neurochemistry, Institute of Neuroscience and Physiology, Sahlgrenska Academy at University of Gothenburg, 43 180 Mölndal, Sweden; 5 Banco de Tejidos de la Fundación CIEN, CIEN Foundation, Carlos III Institute of Health, Alzheimer Center Reina Sofia Foundation, 28006 Madrid, Spain; 6 Memory Clinic, Neurology Service, Hospital General Universitario de Elche, Elche, 03203, Spain; 7 Institut de Neurociències, Departament Bioquímica i Biologia Molecular, Universitat Autònoma de Barcelona, 08193 Bellaterra, Spain; 8 Karolinska Institute-Alzheimer Disease Research center, 171 77 Stockholm, Sweden

**Keywords:** Alzheimer’s disease, Cerebrospinal fluid, Diagnostic, Biomarker, Presenilin 1

## Abstract

**Background:**

Presenilin-1 (PS1) is the active component of the amyloid precursor protein cleaving γ-secretase complex. PS1 protein is a transmembrane protein containing multiple hydrophobic regions which presence in cerebrospinal fluid (CSF) has not been measured to date. This study assesses whether PS1 and other components of the γ-secretase complex are present in CSF.

**Results:**

Here, we show that PS1 is present in ventricular *post-mortem* and lumbar *ante-mortem* CSF, and plasma as 100–150-kDa hetero-complexes containing both the N- and C-terminal fragments (NTF and CTF) of the protein. Immunoprecipitation and immunoblotting with different antibodies confirmed the identity of the PS1 species. The γ-secretase components, APH-1 (anterior pharynx-defective 1) and PEN-2 (presenilin enhancer 2), as well as presenilin-2 (PS2) fragments, co-exist within these CSF complexes, while nicastrin is not detected. These CSF-PS1 complexes differ from active γ-secretase membrane-complexes, and may represent nonspecific aggregation of the PS1 protein. Levels of PS1 complexes are increased in CSF samples from autopsy-confirmed Alzheimer’s disease (AD) cases and were found to be more stable than complexes in CSF from control subjects. Despite similar levels of total PS1 in CSF from probable AD patients and cognitively normal subjects, an increased proportion of highly stable PS1 complexes were observed in AD CSF.

**Conclusions:**

Our data suggest that fragments of the PS1 protein present in CSF as complexes may be useful as a biomarker for AD.

## Background

The major pathological hallmarks of Alzheimer’s disease (AD), the β-amyloid peptide (Aβ) and the abnormally hyperphosphorylated protein tau (P-tau), are also recognized biomarkers which have been extensively investigated (for a review see [1]). However, there is a continuing search for new cerebrospinal fluid (CSF) biomarkers for use in clinical diagnosis, especially for the early stages of AD, and for use in clinical trials.

Other logical AD biomarker candidates are proteins involved in the pathological processing of the large transmembrane protein, the amyloid precursor protein (APP), leading to β-amyloid formation in the AD brain. The Aβ polypeptide is generated by processing of APP, through the successive action of two proteolytic enzymes, β-secretase and γ-secretase [2]. The major neuronal β-secretase has been identified as beta-site APP cleaving enzyme 1 (BACE1; [3]). As BACE1 contains a single transmembrane domain, its presence in CSF is not unexpected [4]. In contrast, γ-secretase consists of four essential subunits: presenilin-1 (or presenilin-2), nicastrin, APH-1 (anterior pharynx-defective 1), and PEN-2 (presenilin enhancer 2) [5]. Presenilin-1 (PS1), a transmembrane aspartyl protease, is the catalytic subunit, and has a nine-transmembrane domain topology [6]. A close homologue of PS1, presenilin-2 (PS2), shares a high degree of homology with the PS1 protein, has functional redundancy and also contains the active site of γ-secretase, forming similar but independent complexes [7].

To our knowledge, the presence of presenilins in CSF has not been reported to date. In this study we investigated if PS1 is detectable in CSF and if altered levels of this protein reflect the pathological condition.

## Results

### PS1 NTF and CTF are present in high molecular mass complexes in CSF and plasma

**Figure 1:**
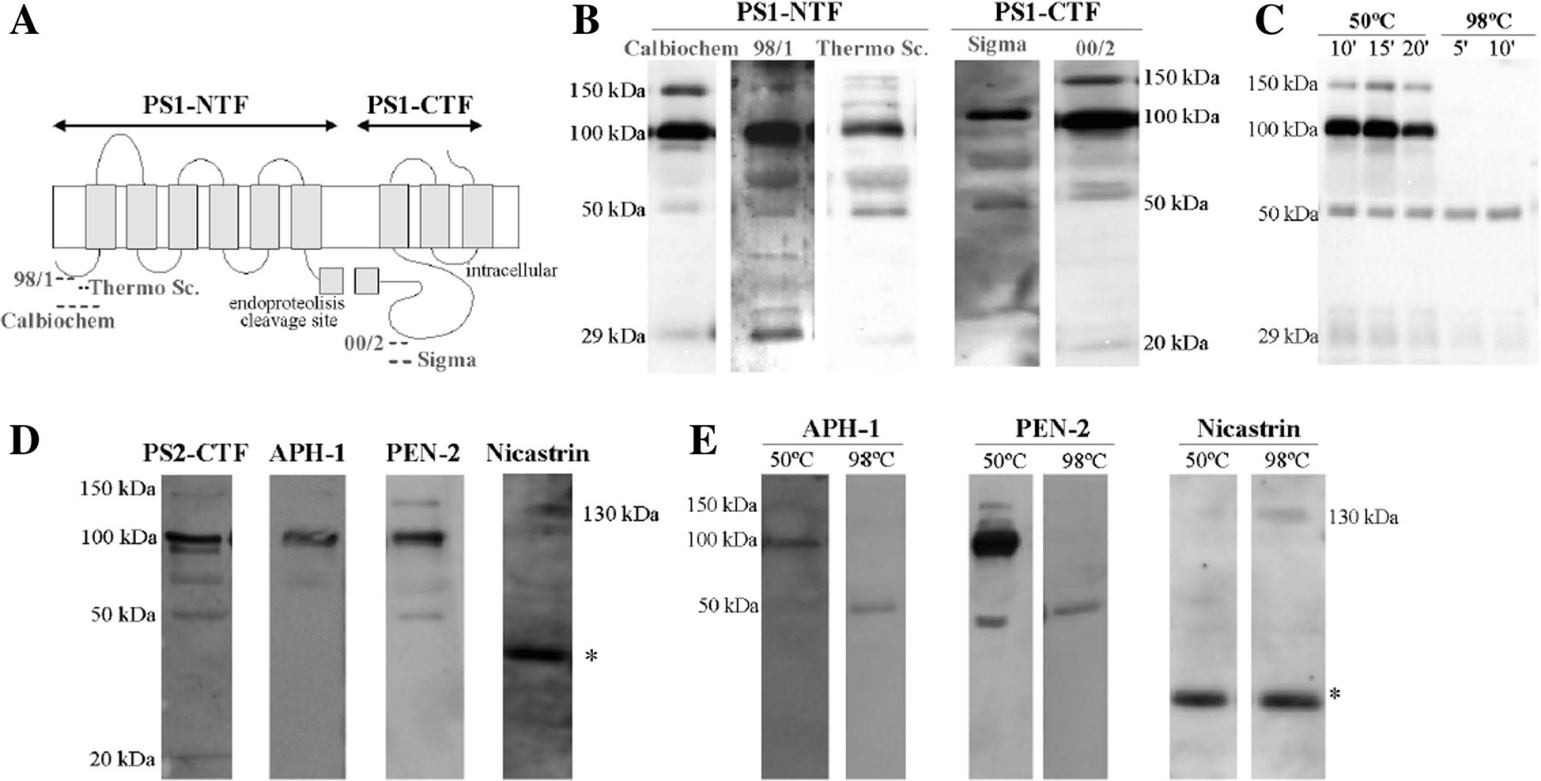
**PS1 complexes are detected in human CSF with alternative antibodies. (A)** Schematic representation of PS1 with epitopes for the anti-PS1 antibodies indicated. **(B)** Human ventricular post-mortem CSF samples from non-demented controls (NDC) were blotted under denaturing conditions with the indicated anti-PS1 antibodies. **(C)** Loss of CSF-PS1 reactivity in CSF samples heated at 98°C compared to 50°C using the NTF-PS1 antibody from Calbiochem. The stability of CSF-PS1 is also affected after 20 minutes of heating at 50°C resulting in loss of immunoreactivity. **(D)** CSF samples were also blotted for the homologue PS2 with an anti-CTF antibody (00/12) and for other subunits of the γ-secretase complexes, APH-1, PEN-2 and nicastrin. Nicastrin was the only γ-secretase partner with a different banding pattern, with a 130-kDa band corresponding to the molecular mass of the protein, which was weakly detectable in some of the CSF samples tested. The ~35-kDa band (*) may correspond to nonspecific binding by the antibody, as fragments of this size have not been previously demonstrated to be positive for nicastrin. **(E)** Effect of denaturation temperature prior to electrophoresis (heating for 5 min at 98°C or 10 min at 50°C) on stability of APH-1, PEN-2 and nicastrin. High molecular mass complexes of APH-1 and PEN-2 were only detected after heating at 50°C, while the 130 kDa-kDa nicastrin subunit was detected after denaturation at 98°C, indicating that nicastrin is not a part of the complexes. Illustrative examples (from 3 different experiments).

PS1 is known to undergo endoproteolytic cleavage as part of its maturation, generating N- and C-terminal fragments (NTF and CTF) [8], with very little full-length PS1 detectable in brain or cultured cells [9]. To determine the presence of PS1 in human CSF, we first examined ventricular *post-mortem* CSF samples by Western blotting using different anti-PS1 NTF and CTF antibodies (a schematic representation of PS1 structure and epitopes for antibodies is represented in Figure 1A). Immunoblotting, using an anti-PS1 NTF antibody (Calbiochem), revealed predominant bands of approximately 100 and 150 kDa and only a faint 29-kDa band corresponding to PS1-NTF (Figure 1B). Immunoblotting with the PS1-CTF antibody 00/2 detected the 100–150-kDa complex, and a weak ~20-kDa band corresponding to PS1-CTF (Figure 1B). The use of alternative NTF and CTF antibodies confirmed the specificity of the PS1 signal in CSF samples (Figure 1B). Similar high molecular mass complexes of PS1, composed at least in part of both NTF and CTF, have been previously described in untransfected cells [10]. A similar banding pattern was obtained for PS2 complexes in CSF using the PS2-CTF antibody 00/12 (Figure 1C); PS2 fragments having similar high molecular mass as PS1 [7,11].

Difference in assay conditions, such as the presence of detergents, can affect the molecular interactions necessary for PS1 complex formation and its subsequent detection [10]. PS1 aggregates have previously been described as temperature-sensitive [12]. As the denaturation temperature prior to electrophoresis is not standardized, we determined the effect of sample heating during preparation on the stability of high molecular mass PS1 complexes. The high temperature used during sample preparation for electrophoresis (98°C compared with 50°C), resulted in an overall loss of PS1 immunoreactivity, the higher molecular mass complexes being the most severely affected (Figure 1C). These experiments demonstrate that different assay methods and sample handling may influence the stability and detection of PS1 complexes. Studies performed with samples denatured at 98°C can therefore underestimate and fail to detect PS1 complexes. In this study, all analyses performed on PS1 avoided freeze-thaw cycles and denaturation before electrophoresis was conducted at 50°C.

Interestingly, under the conditions used for PS1 complex detection, PEN-2 and APH-1 immunoreactivities in CSF displayed similar electrophoretic banding patterns. However the traces of nicastrin that were detected were not part of the high molecular mass presenilin complexes (Figure 1D). Nicastrin was only reliably detected when sample preparation for electrophoresis was performed at 98°C (Figure 1E).

**Figure 2:**
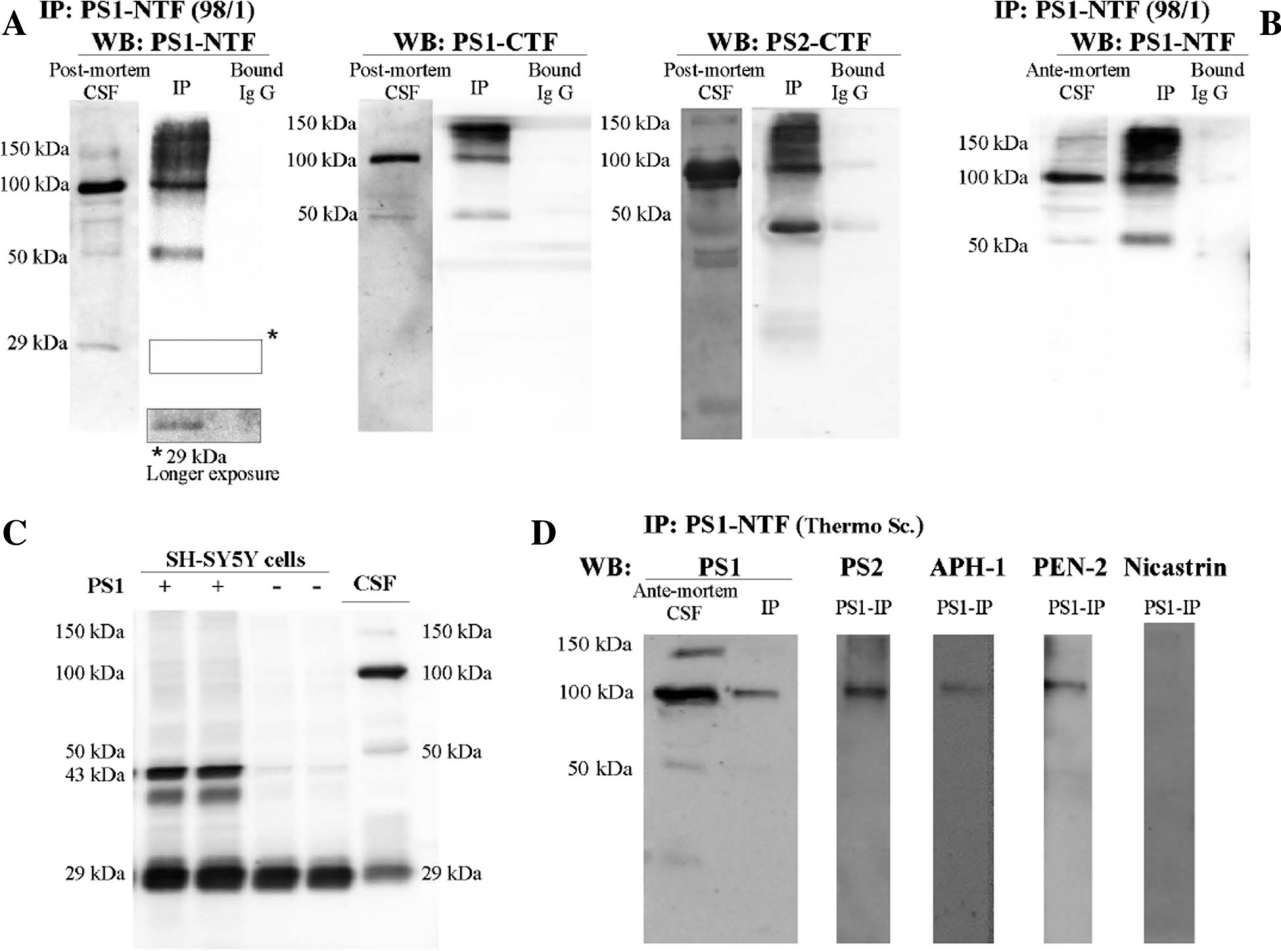
**Immunoprecipitation of PS1 in human CSF. (A)** Ventricular *post-mortem* CSF was immunoprecipitated with the anti-PS1 98/1 antibody, and precipitated proteins (IP) were immunoblotted with PS1-NTF (Calbiochem) or PS1-CTF antibodies (00/2). Precipitated proteins (IP) were immunoblotted with the PS2-CTF antibody 00/12. **(B)** Lumbar *ante-mortem* CSF was immunoprecipitated with NTF-PS1 antibody 98/1 or with antibody from Thermo Scientific, and precipitated proteins (IP) detected with an NTF-PS1 antibody from Calbiochem. **(C)** Extracts from SH-SY5Y cells over-expressing PS1 (+) and extracted in the presence of 1% Nonidet P-40/0.5% Triton X-100 were run in parallel with *post mortem* CSF samples from non-diseased control subjects. The presence of a ~43 kDa PS1 holoprotein in membrane extracts was detected with the NTF-PS1 antibody from Calbiochem. Comparison of the immunoreactive bands indicates that this 50-kDa band is not the PS1 holoprotein. **(D)** PS1-precipitated proteins (PS1-IP) were also immunoblotted with anti APH-1, PEN-2 or nicastrin antibodies. APH-1 and PEN-2, but not nicastrin were detected in PS1 immunoprecipitates. Extracts incubated with protein A-Sepharose with an irrelevant rabbit IgG (Bound IgG), were analyzed in parallel as negative controls.

To further examine the identity of PS1complexes in human CSF, we performed immunoprecipitation/Western blot analysis (Figure 2A). *Post-mortem* CSF samples were immunoprecipitated using the 98/1 antibody, an alternative PS1-NTF antibody which is effective in immunoprecipitating human PS1 [13]. Western blot analysis with the PS1-NTF antibody from Calbiochem and the PS1-CTF antibody 00/2 detected the predominant 100–150-kDa complexes. These bands were not observed in negative immunoprecipitation controls, including when an irrelevant rabbit IgG was used (Figure 2A). Considerable amounts of the 50-kDa species were detected in PS1 immunoprecipitates using both anti-NTF and CTF antibodies. This species was also detected in NTF-PS1 immunoprecipitates from freshly collected *ante-mortem* lumbar CSF samples (Figure 2B). These NTF/CTF-PS1 heterodimers probably arise from the 100 and 150 kDa complexes during elution at acidic pH, and are therefore not likely to be *post-mortem* artefact. The 50-kDa PS1 may also be derived from NTF and CTF-PS1 aggregation, as the holoprotein extracted from SH-SY5Y cells transfected with the human PS1 cDNA had a mass of ~43 kDa (Figure 2C).

The other known subunits of the γ-secretase complex, APH-1 and PEN-2, were detected in freshly collected lumbar samples immunoprecipitated with an alternative PS1-NTF antibody, indicating that these components are part of the complexes (Figure 2D). These PS1 complexes from both *post mortem* and freshly collected lumbar CSF also contain PS2 fragments, demonstrating the existence of mixed complexes of PS1 and PS2 in the CSF (Figure 2A, 2D).

**Figure 3:**
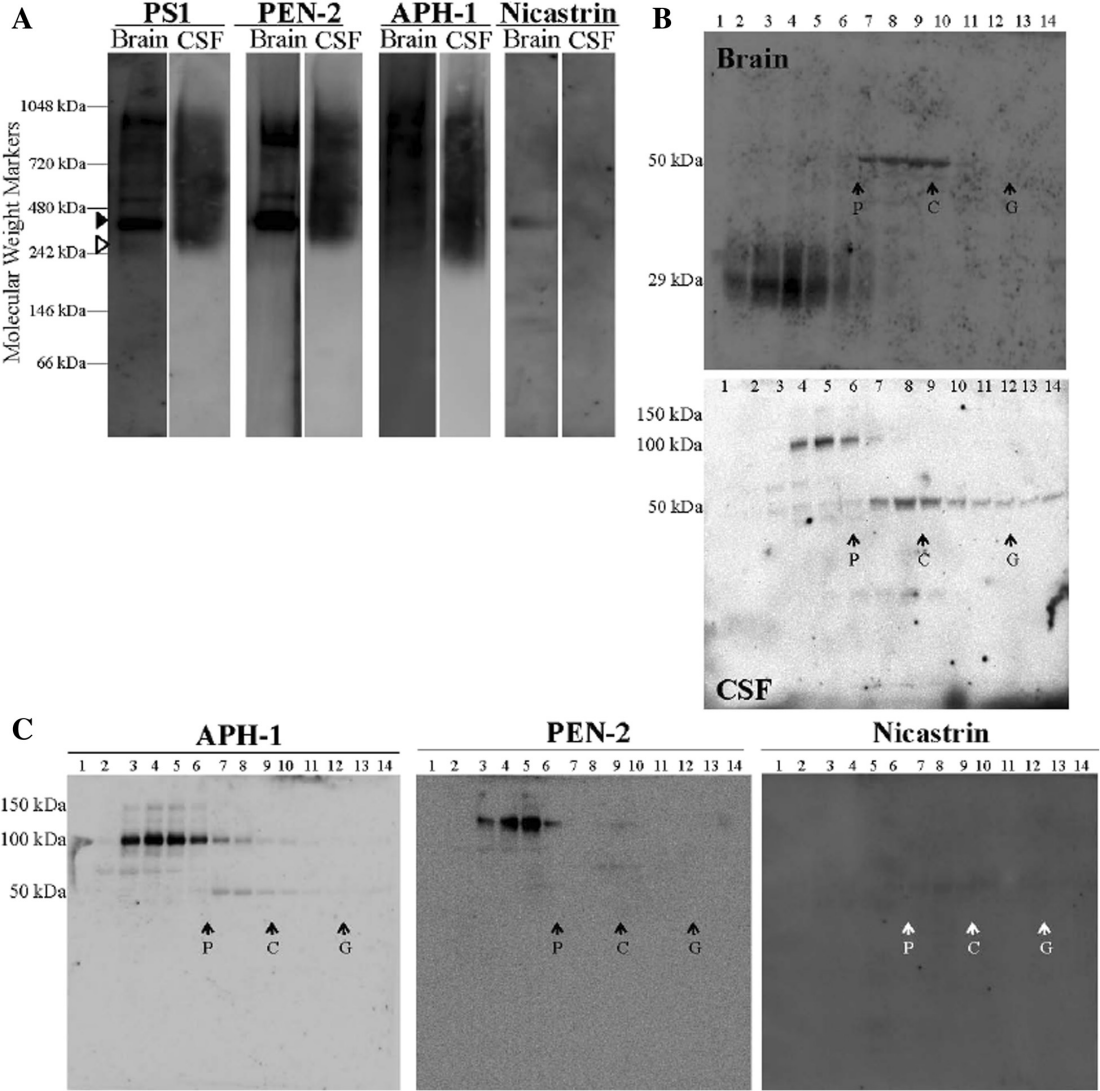
**Characterization of CSF-PS1 complexes. (A)** Brain γ-secretase complexes (frontal cortex from non-diseased control subjects extracted in buffer containing 0.5% dodecylmaltoside) were analyzed by blue native-PAGE and compared with PS1 complexes isolated from lumbar CSF samples (NDC cases). Incubation of blots with antibodies for the different γ-secretase subunits confirmed that nicastrin is not present in CSF-PS1 complexes. **(B)** The same brain extract and CSF samples were also fractionated on 5-20% sucrose density gradients. The fractions (collected from the top of each tube) were immunoblotted for NTF-PS1 under denaturing conditions with the antibody from Calbiochem. Enzymes of known sedimentation coefficient, β-galactosidase (G, 16.0S; ~540 kDa), catalase (C, 11.4S; ~232 kDa) and alkaline phosphatase (P, 6.1S; ~140-160 kDa) were used as internal markers. **(C)** CSF fractions from sucrose gradients were also blotted for APH-1, PEN-2 and nicastrin. Nicastrin was mostly undetectable in fractionated CSF samples.

The CSF-PS1 complexes were further characterized and compared to human brain γ-secretase membrane complexes extracted in assay buffer containing 0.5% dodecylmaltoside [14]. Both CSF and brain extracts were analyzed by blue native-PAGE (Figure 3A). As expected, in dodecylmaltoside-solubilized brain membrane fractions, all four γ-secretase subunits co-migrated in several bands. A weak band immunoreactive for PS1 was identified at ~250 kDa (open arrowhead). This molecular mass corresponds to the smallest sized complex in which active γ-secretase has been observed [15]. However, this band was not immunoreactive for all the other γ-secretase subunits. A predominant immunoreactive band, with a molecular mass of ~450 kDa (closed arrowhead), was detected with all antibodies. Other high molecular mass bands, corresponding to large γ-secretase complexes [15,16], were also immunoreactive for the different γ-secretase subunits, PS1, APH-1 and PEN-2. By blue native-PAGE, PS1-CSF complexes appeared as a smear from ~200 to 1000 kDa (Figure 3A). In agreement with results obtained from SDS-PAGE analysis; only APH-1 and PEN-2 were present in these CSF-PS1 complexes (Figure 3A). The interpretation of results from blue native-PAGE analysis of membrane protein complexes is tricky, especially for the limitations of these methods of size determination by comparison with the mobilities of hydrophilic molecular mass markers.

Previous studies have also utilized gradient centrifugation to characterize PS1 complexes [10]. PS1 complexes were further characterized in lumbar CSF samples and dodecylmaltoside-solubilized brain membrane fractions by fractionation on sucrose density gradients containing the detergent Brij 97 (Figure 3B).

The majority of the PS1 solubilized from human brain in presence of dodecylmaltoside accumulated close to the alkaline phosphatase marker (molecular mass ~140-160 kDa), and were resolved as a 29-kDa band by Western blotting using an NTF-PS1 antibody under denaturing conditions. Brain-PS1 complexes were also identified in denser fractions, close to the sedimentation rate of catalase (molecular mass ~232 kDa). Interestingly, these large PS1 complexes were resolved as ~50-kDa heterodimers. However, CSF-PS1 complexes which sediment close to alkaline phosphatase were resolved as stable 100-150 kDa complexes, instead of monomeric NTF-PS1 (Figure 3B). Large CSF-PS1 complexes were also resolved as ~50-kDa heterodimers, indicating that these complexes are less stable in the CSF than those which sediment at 100-150 kDa. APH-1 and PEN-2 were again identified as components of the CSF-PS1 complexes (Figure 3C).

No measurable γ-secretase activity [17] was detected in freshly collected lumbar CSF samples from non-demented subjects (n = 5). These results indicate that CSF-PS1 complexes differ from the active γ-secretase complexes found in the brain, and may represent nonspecific aggregation of PS1 fragments (together with APH-1 and PEN-2, but not nicastrin) in the CSF.

**Figure 4:**
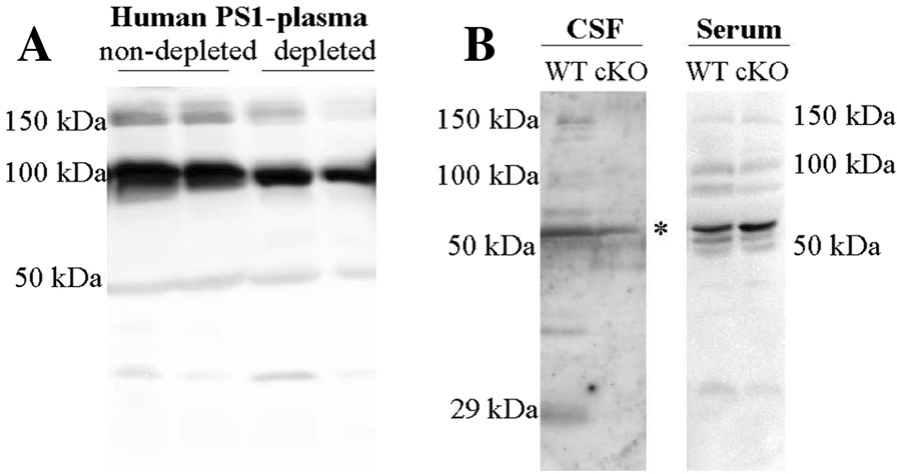
**PS1 complexes are present in human and mouse CSF and plasma. (A)** Plasma samples from NDC subjects were blotted with an anti-NTF-PS1 antibody (from Calbiochem) with and without depletion of plasma abundant proteins by immunoaffinity-based protein subtraction chromatography with IgY microbeads (Seppro™). **(B)** CSF and plasma samples from control wild-type (WT) and PS1 conditional knockout mice (cKO) were blotted with an anti-NTF-PS1 antibody. In general, immunoreactivities of the bands corresponding to PS1 complexes were weak in mouse (or rat, not shown) than in human samples. PS1 levels are reduced in CSF from *PS1* cKO mice, and unchanged in plasma. PS1 immunoreactivity detected in CSF from *PS1* cKO mice may be due to PS1 expressed in glia and interneurons. However, it is also highly likely that some nonspecific binding may occur at 50 kDa (*).

Soluble PS1 complexes were also detected in human plasma (Figure 4A), and in mouse CSF and plasma (Figure 4B). PS1 complexes were mostly absent in CSF from a *PS1* cKO mouse model where PS1 expression has been selectively eliminated in neurons from the postnatal forebrain, decreasing brain PS1 levels [18]. However, PS1 complexes were present in serum samples from these *PS1* cKO mice, indicating that CSF and plasma PS1 complexes have distinct cellular origins.

### PS1 levels are increased in AD ventricular *post-mortem* CSF

**Figure 5:**
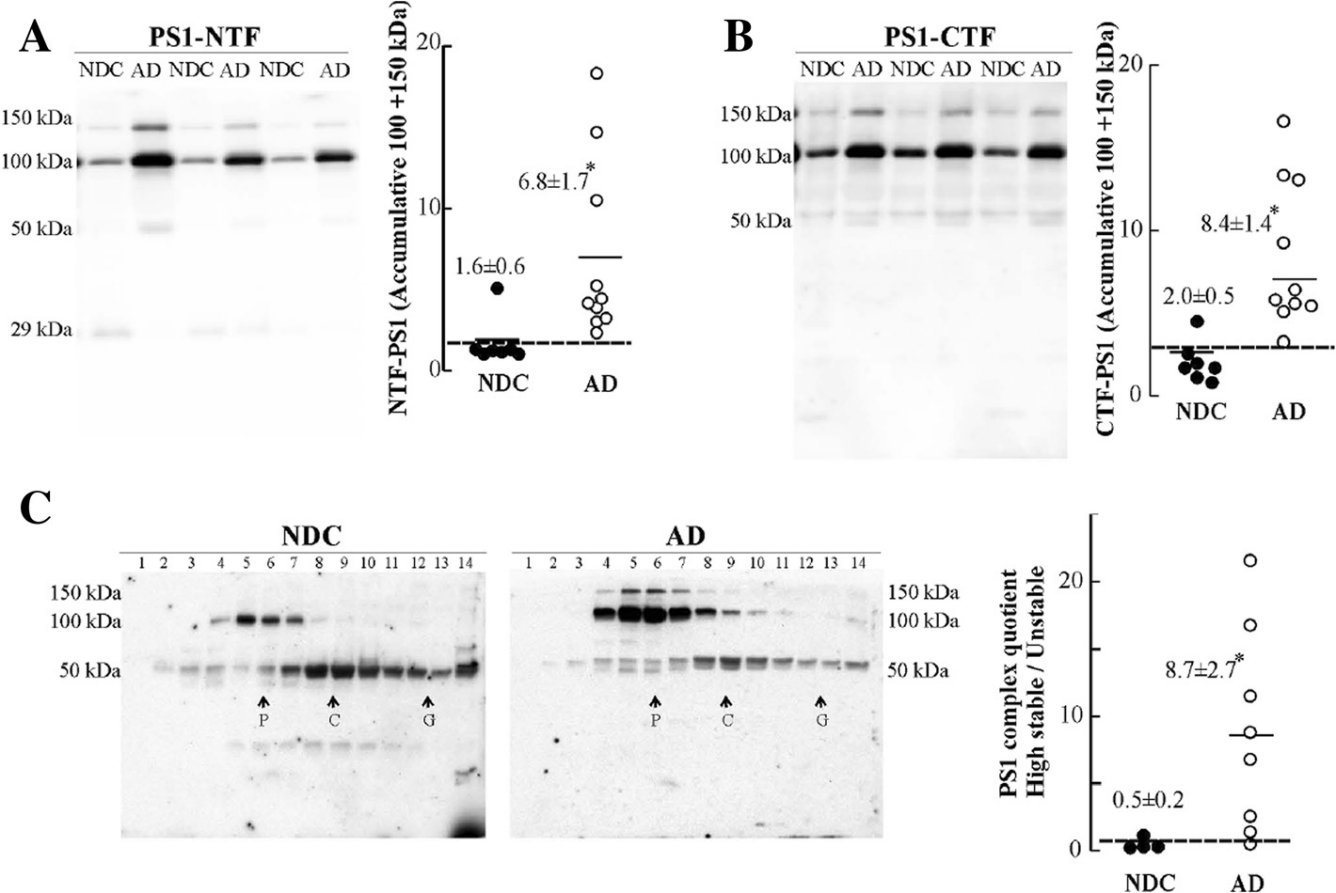
**PS1 stable complexes are increased in AD *post-mortem *CSF. (A)** Representative blot of NTF-PS1 in *post-mortem* CSF samples from 10 AD (open circles) and 7 NDC controls (closed circles). The densitometric quantification of the accumulative immunoreactivity from the sum of the higher molecular mass PS1 complex (100 + 150 kDa) is shown. **(B)** Immunodetection and densitometric quantification of higher molecular mass PS1 complex (100 + 150 kDa) from CSF samples blotted with an anti-CTF-PS1 antibody. Dashed lines represent arbitrary cutoffs that maximally discriminated between AD and NDC groups. **(C)** PS1 complexes in 4 (of 7) NDC (closed circles) and 8 (of 10) AD CSF samples (open circles) were fractionated on 5-20% sucrose density gradients. The fractions (collected from the top of each tube) were immunoblotted for NTF-PS1 under denaturing conditions with the antibody from Calbiochem. Enzymes of known sedimentation coefficient, β-galactosidase (G, 16.0S; ~540 kDa), catalase (C, 11.4S; ~232 kDa) and alkaline phosphatase (P, 6.1S; ~140-160 kDa) were used as internal markers. Representative blots are shown. A quotient between highly stable complexes (100 + 150 kDa immunoreactive bands sediment closer to alkaline phosphatase, fractions 2-7) and unstable complexes (50-kDa immunoreactive bands sediment closer to catalase, fractions 8-12) was defined and represented. Dashed lines represent an arbitrary cutoff that maximally discriminated between AD and NDC groups. The data represent the means (in arbitrary units) ± SEM. *Significantly different (*p* < 0.05) from the NDC group, as assessed by the Mann-Whitney *U* test.

To assess whether PS1 levels are altered in AD, we analyzed ventricular *post-mortem* CSF for NTF and CTF-PS1 in 10 AD and in 7 NDC samples. The 100 and 150-kDa complexes were detected with an anti-NTF antibody in all CSF analyzed (Figure 5A). The immunoreactivity for the 100 + 150 kDa complexes increased by ~325% in AD compared to NDC subjects (*p* = 0.005). New aliquots of the same CSF samples were immunoblotted with an anti–CTF-PS1 antibody (Figure 5B). In good agreement, CTF-PS1 immunoreactivity for the 100 + 150 kDa complexes increased by ~320% in AD compared to NDC subjects (*p* = 0.002). The minor form of PS1 (29-kDa) was decreased in AD compared to NDC subjects (~70%; *p* = 0.03); while the 20 kDa-band corresponding to monomeric PS1-CTF was only very weakly detected and not quantified.

Fractionation on sucrose density gradients followed by Western blotting under denaturing conditions revealed that the highly stable 100-150 kDa complexes were more abundant in AD samples, while less stable complexes that sediment in denser fractions were mostly detected in NDC samples. This difference enabled us to define a quotient between highly stable complexes (represented by 100–150-kDa heterodimers which sediment closer to alkaline phosphatase) and unstable complexes (50-kDa complexes which sediment closer to catalase), allowing us to fully discriminate between the two groups (Figure 5C).

### Highly stable PS1 complexes are increased in AD lumbar *ante-mortem* CSF

**Figure 6:**
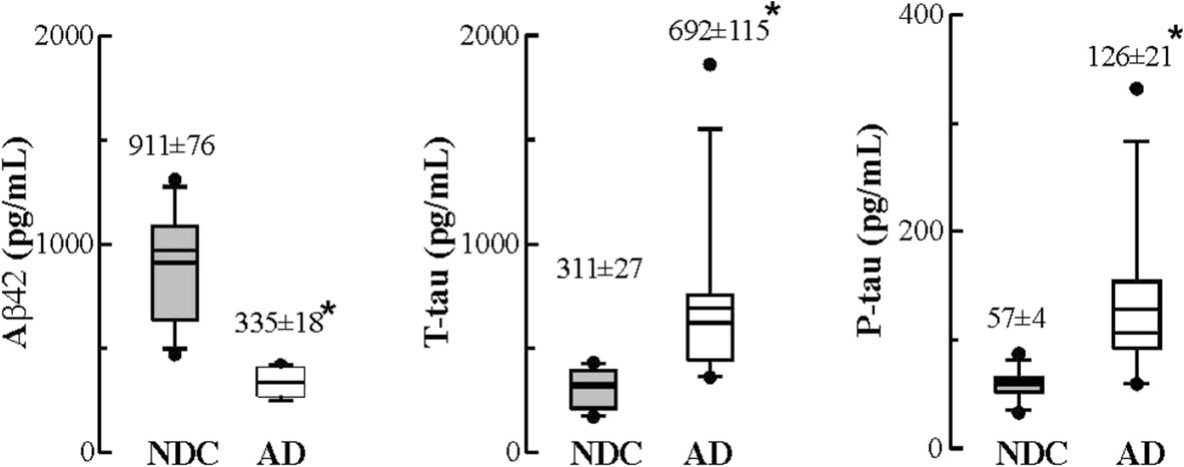
**Levels of classical AD biomarkers in lumbar CSF.** Box plot of CSF levels of Aβ42, T-tau and P-tau for the 12 probable AD cases (open circles) and the 12 NDC controls (closed circles). Means ± SEM are shown. Mann-Whitney *U* test, *p < 0.05, **p < 0.01.

**Figure 7:**
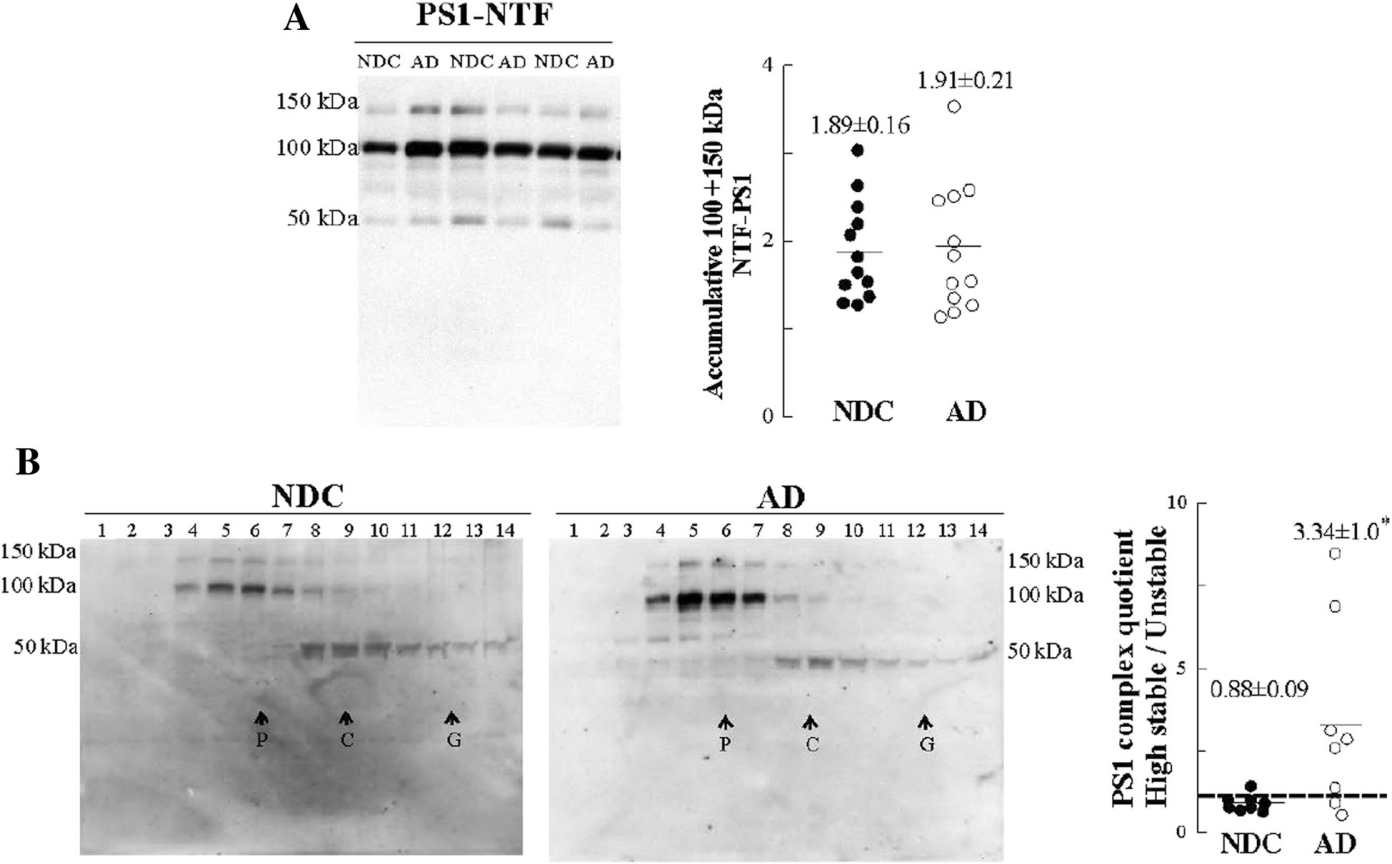
**Stable PS1 complexes are increased in AD *ante-mortem *CSF. (A)** Representative blot and densitometric quantification of the accumulative immunoreactivity from the sum of higher molecular mass PS1 complex (100 + 150 kDa) in lumbar CSF samples from 12 AD (open circles) and 12 NDC controls (closed circles). **(B)** Eight of the 12 cases available from both the AD and NDC groups were also fractioned into 5-20% sucrose density gradients, and blotted with the NTF-PS1 antibody under denaturing conditions. Internal markers were β-galactosidase (G), catalase (C) and alkaline phosphatase (P), as in Figure 5. In the right panel the individual values for the quotient between highly stable complexes (100 + 150 kDa immunoreactive bands sediment at fractions 2-7) and unstable complexes (50 kDa immunoreactive bands sediment at fractions 8-12) are shown. *Significantly different (*p* < 0.05) from the NDC group, as assessed by Mann-Whitney *U* test.

We have demonstrated that high molecular mass PS1 complexes are increased in post-mortem AD CSF. These PS1 complexes were also assessed in lumbar CSF from probable AD and control subjects. Measurement of classical AD biomarkers displayed elevated CSF T-tau and P-tau and low levels of Aβ42 in probable AD samples (Figure 6). As expected, predominant PS1 bands of approximately 100 and 150 kDa were detected in all cases, although no notable changes were observed in total PS1 levels between probable AD and NDC subjects (Figure 7A). No detectable amount of the 29-kDa species was evident in lumbar CSF samples, nor in immunoprecipitates from lumbar CSF samples (see Figure 2). Sucrose density centrifugation profiles (determined in 8 of the 12 lumbar CSF cases, for both the AD and NDC groups, due to limited volumes) revealed a major contribution of the highly stable PS1 complexes in probable AD cases, compared to NDCs (Figure 7B). Interestingly, the quotient of the PS1 complexes fractionated in the sucrose gradient correlates with Aβ42 (r = 0.51, *p* = 0.04) and with P-tau levels (r = 0.57, *p* = 0.02). In the NDC group, age trends to positive correlates with the quotient of the PS1 complexes (n = 8; r = 0.70, *p* = 0.07).

### Levels of full-length BACE1 in CSF

**Figure 8:**
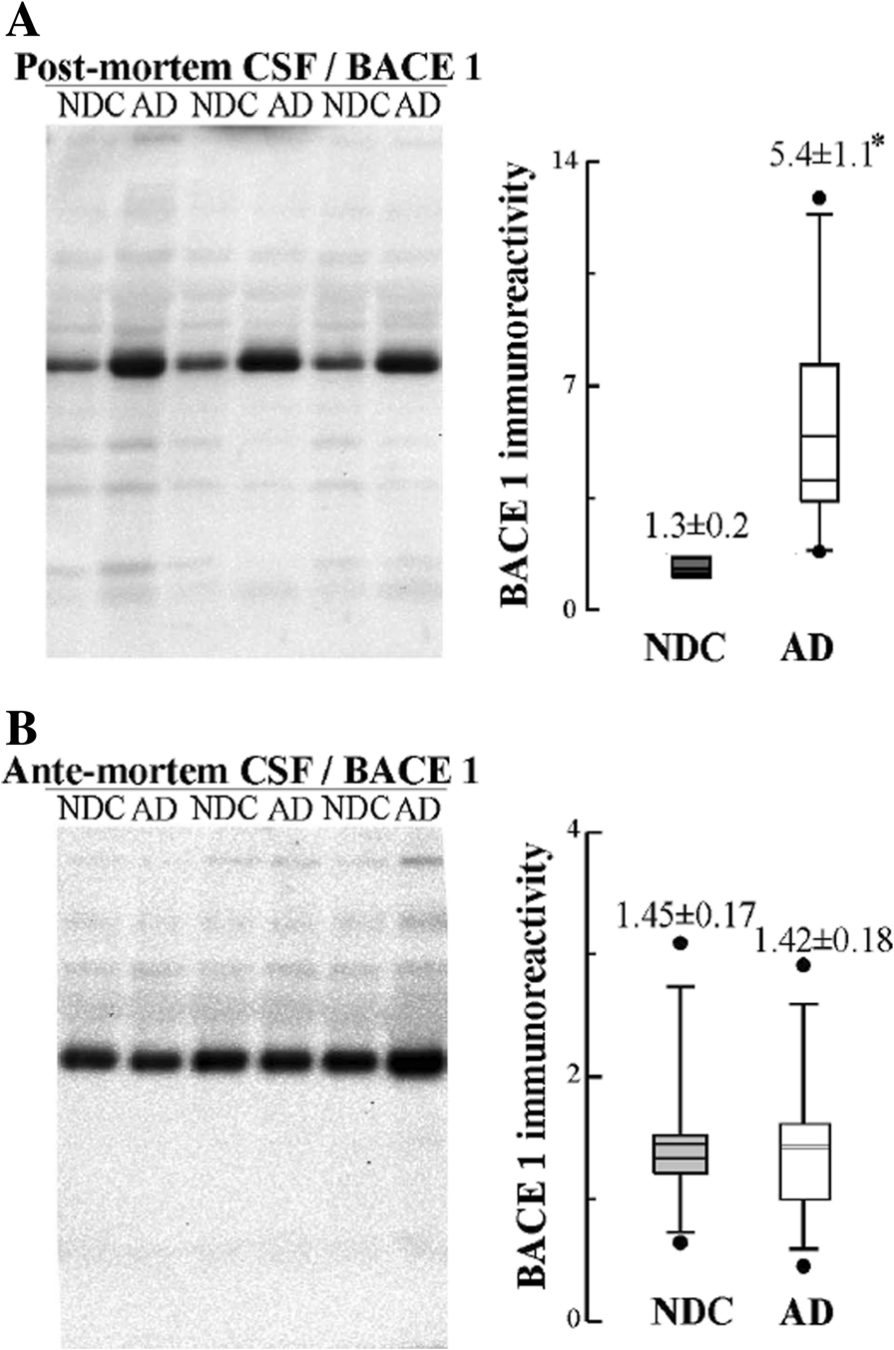
**BACE1 levels in the *post-mortem *and *ante-mortem *CSF samples. (A)** Immunodetection and densitometric quantification of the ~70-kDa immunoreactive BACE1 band from the same *post-mortem* CSF cases, AD (open circles) and NDC (closed circles), presented in Figure 5; and **(B)** from the same *ante-mortem* CSF cases, AD (open circles) and NDC (closed circles), presented in Figure 6. The box blot of BACE1 levels includes the means of the immunoreactivities (in arbitrary units) ± SEM (determinations by duplicate). *p < 0.05 significantly different from NDC group, as assessed by the Student’s *t* test.

BACE1, the major neuronal β-secretase, together with γ-secretase, processes APP through the amyloidogenic pathway [2]. BACE1 activity levels have been found to increase in AD CSF and it has been identified as a potential diagnostic marker for AD [4,19-21]. It’s has been assumed that the BACE1 present in CSF is a truncated soluble form of the originally membrane-bound BACE1, missing the transmembrane and intracellular domains [22]. However, it’s possible to identify BACE1 in CSF by Western blotting using an anti-BACE1 antibody which binds the C-terminal intracellular domain (antibody D10E5 from Cell Signaling, with antigenic determinant located in residues surrounding Asp^490^ of human BACE1 protein). Using this antibody, we have evaluated BACE1 levels by Western blotting in the same set of *post-mortem* CSFs. Full-length BACE1 was significantly elevated in AD patients compared with controls (*p* = 0.018; Figure 8A). Full-length BACE1 levels also correlated with PS1 when the quotient of the PS1 complexes fractionated on sucrose gradients are considered for all samples (r = 0.95, *p* < 0.001), or for AD cases (r = 0.93, *p* < 0.001). No change was observed in full-length BACE1 levels evaluated in lumbar CSF from probable AD and control subjects (Figure 8B).

## Discussion

To our knowledge, the possibility that PS1 can be assessed in the CSF has thus far not been considered. Here, we demonstrate that heterodimeric complexes composed of NTF and CTF PS1 are detectable in CSF. To date, the presence of soluble CTF-PS1 has only been demonstrated in culture medium of primary neurons and HEK cells [23].

The majority of mature brain PS1 protein is present as proteolytic NTF- and CTF-PS1 fragments [8]. The existence of 100–150-kDa complexes containing both endogenous NTF and CTF-PS1 has only previously been described in cultured cells [10]. PS1 exhibiting higher molecular mass bands in SDS-PAGE, has also been described in cellular models over-expressing the protein [24,25]. These high molecular mass bands of PS1 were detected by alternative anti-NTF and CTF-PS1 antibodies, confirming that heterodimers contain both PS1 fragments. Triton X-100, a common detergent frequently used to extract and solubilise membrane bound proteins, disassembles the 100–150-kDa complexes [10], and these PS1 heterodimeric species have therefore been mischaracterized in the many previous studies. The sensitivity of these complexes to high temperatures during sample preparation may contribute to its mischaracterization. The presence of the minor 29-kDa NTF and 20-kDa CTF species was only clearly detectable in *post-mortem* CSF, where artefacts are likely to appear. Similarly, the 50-kDa species was only resolved as a major PS1 band in immunoprecipitates after elution at acid pH.

Ultracentrifugation in sucrose density gradients containing the detergent Brij 97 confirms the existence of stable complexes of 100–150-kDa, and also of large complexes which sediment in regions closer to 200 and 250 kDa. These large complexes are unstable and resolve as 50-kDa components during electrophoresis under denaturing conditions.

γ-Secretase exists on the plasma membrane as an intact complex composed of its subunit components [15,26], at a stoichiometry of presenilin:PEN-2:nicastrin:APH-1, 1:1:1:1 [27]. However, PS1 can associate intra-molecularly to form higher order complexes [28], where nicastrin, APH-1 and PEN-2 do not seem to be required for its hetero- and homodimerization [29]. Interestingly, our studies have identified APH-1 and PEN-2, but not nicastrin in the PS1-complexes. γ-Secretase activity depends on the presence of all four components of the complex, including nicastrin [30,31]. As we were unable to detect γ-secretase activity in human CSF, PS1 complexes in CSF are therefore likely to be the result of nonspecific aggregation of the protein. PS1-CSF complexes appeared as a smear (from ~200 to 1000 kDa) and not as defined complexes on blue native-PAGE. PS2 has been shown to form similar but independent γ-secretase complexes in cell membranes, and PS2 does not co-precipitate with PS1 fragments in membrane extracts [7,32]. We were able to demonstrate that fragments of PS2 are bound to the same PS1 complexes in CSF. As presenilins are proteins with large numbers of hydrophobic regions, presenilin fragments may be highly unstable in CSF, and spontaneously form complexes. Indeed, like many membrane proteins, PS1 has exhibited a tendency to aggregate under non-native conditions [11,12]. Thus, in normal conditions, the amount of free CTF and NTF in CSF should be very low as PS1 predominantly exists as complexes. Preliminary analysis indicates that PS1 complexes do not contain some of the abundant CSF proteins known to bind hydrophobic proteins (e.g., albumin). Further characterization of these complexes is yet to be conducted. The possibility that other proteins, such as Aβ, may also be part of the PS1 complex needs to be addressed. Similarly, the source and mechanism of how PS1 appears in CSF is yet to be determined. Active secretion is unlikely, and it is still unclear if passive release from brain cells or neuronal death may be major contributing factors, as recently observed for BACE1 [33].

Stable 100–150-kDa complexes are more abundant in AD CSF, and this may reflect differences in the hydrophobic and ionic properties of PS1 complexes formed under amyloidogenic conditions. In this context, it is also remarkable that the 29-kDa NTF, detectable only in *post-mortem* CSF, is less abundant in AD samples, indicating that complexes formed under amyloidogenic conditions are particularly stable. Whether PS1 complexes differ between AD and non-demented subjects requires further research.

It is likely that molecular aberrations in the AD brain are reflected in the CSF. Although it cannot be excluded that the accumulation of Aβ in AD may be partly due to deficient clearance of the peptide, an increase in the generation of Aβ is plausible. Thus, an increase in activity and/or levels of β- and γ-secretase protein components should be expected. These catalytic effectors of β- and γ-secretase are key enzymes in pathological amyloid processing and the subsequent generation of Aβ constitutes a central event in AD progression. However, it is still unclear if γ-secretase activity is altered. Reported levels of PS1 in AD brains have been contradictory. Reports have displayed an increase [34,35], unchanged levels [36] or even a decrease [37,38] in levels, compared to levels in non-demented brains. At the transcriptional level, early reports indicate no differences between PS1 mRNA levels in AD brain compared to controls [39]. However subsequent research suggests that PS1 mRNA levels in human AD brains are significantly higher than in those with no dementia [34,40]. Interestingly, we have shown that Aβ peptide treatment of cultured cells is able to induce increases in cellular PS1 levels [41], probably as part of a vicious cycle of Aβ generation.

In our analysis, ventricular *post-mortem* samples display large differences in the level of PS1 and the stability of its complexes. Lumbar CSF samples only display differences in PS1 complex stability as resolved by sucrose density gradients. Thus, despite high levels of PS1 correlating with an increased proportion of stable complexes in *post-mortem* CSF, results in lumbar CSF suggest that the early and more significant phenomenon is the change in the dynamics of the assembly of PS1 complexes, a change that appears to be a better marker for discriminating between pathological samples than total PS1 protein levels alone. In any case, the comparison between the two different sets of CSF samples is difficult, as the potential existence of artefacts in the *post-mortem* samples and the possibility that ventricular and lumbar CSF samples may differently reflect brain protein content. Moreover, our collection of lumbar CSF samples display clear differences for classical AD biomarkers, but the inherent uncertainty in clinical diagnosis should indeed be considered. Although, the more obvious difference between the CSF collections is that *post-mortem* CSF reflects late stages of the disease, whereas lumbar CSF corresponds to earlier stages of disease in patients. Nonetheless, CSF PS1, combined with other biomarkers, may constitute a potential new marker for AD. Furthermore, CSF PS1 may have value as a marker of disease progression or for monitoring treatment.

As there is still a lack of reliable blood biomarkers for neurodegenerative disorders, it is important to assess if levels in plasma of new potential biomarkers correlate with AD. PS1 is expressed in many peripheral organs as well as the brain [42,43]. The presence of PS1 complexes in the serum of *PS1* cKO mice, in which PS1 is specifically absent in neurons of the forebrain, while mostly absent in CSF, suggests that plasma and CSF PS1 may have distinct cellular origins. Although a small contribution of brain PS1 to plasma levels cannot be discounted, our results indicate that PS1 detection in plasma will not be an efficient marker for brain disorders.

## Conclusion

In conclusion, accurate diagnosis of AD during life is essential. Numerous laboratories have reported an increase in levels of T-tau and P-tau in CSF, but tau is also increased as result of other neurological processes. While levels of the pathological Aβ42 species are also increased in the AD brain, the levels in CSF are decreased due to increasing deposition [44]. These biomarkers have shown diagnostic accuracy for incipient AD [45], however, there is still a need to identify additional specific biochemical markers of AD. Proteins involved in the pathological processing of amyloid are potential candidates. In this study we demonstrate that CSF PS1 has potential as a new biomarker. The diagnostic value of CSF PS1 in the early stages of AD and in other dementias requires further study. In addition, biomarkers are also important to evaluate drug effects underlying pathophysiology of the disease [44]. One of the promising AD drugs under development are γ-secretase inhibitors [46,47]. Estimation of PS1 CSF levels may therefore be useful to challenge the disease-modifying effects of newer Alzheimer’s therapy.

## Methods

### Patients, PS1 conditional knockout mice and PS1 over-expressing cells

#### ***Post-mortem material***

Ventricular CSF was obtained *post-mortem* at the Banco de Tejidos, Fundación CIEN (Madrid, Spain), Fundación Hospital de Alcorcón (Madrid, Spain) and Fundación AlzheimUr (Murcia, Spain). Samples contaminated with blood were excluded from analysis. CSF was centrifuged at 1000 × g for 15 min to eliminate cells and insoluble material prior to biochemical analyses. AD cases [n = 10 (5 female and 5 male); mean age ± standard error of mean (SEM); 77 ± 2 years] were selected based on clinical history of dementia and neuropathological CERAD diagnosis [48]. Samples from age-matched non-demented control (NDC) cases had no clinical or pathological features of dementia [n = 7 (2 female and 5 male); 72 ± 3 years]. Only samples with a post-mortem interval lower than 14 hr were included, with no differences between groups.

Microsomal membranes were solubilized from 0.2 g of human frontal cortex (two NDC cases from Banco de Tejidos, Fundación CIEN) in buffer containing 0.5% dodecylmaltoside, 20% glycerol, and 25 mM Bis-Tris/HCl, pH 7.0, as previously described [14]. γ-Secretase complexes extracted from membrane preparations were analyzed by blue native-PAGE.

#### ***Clinical material***

Lumbar CSF samples were obtained from 12 patients (5 men and 7 women, 71 ± 5 years) with mild-moderate AD, and 12 healthy volunteers (5 men and 7 women, 66 ± 9 years) from Huddinge University Hospital (Stockholm, Sweden). All AD patients fulfilled the NINCDS-ADRDA criteria for “probable” AD [49]. Controls had no history or symptoms of neurological or psychiatric disorders, or memory complaints, and had an MMSE score of 28 or higher. Five non-demented control CSF samples (2 men and 3 women, 52 ± 7 years) obtained from the Hospital General Universitario de Elche (Elche, Spain) were analytically processed within 24-48 hours of collection to avoid freezing and thawing of the samples.

Plasma samples from five healthy volunteers (76 ± 1 years) were collected in heparinized tubes at the Hospital General Universitario de Elche and separated from whole blood by centrifugation at 3000 × g for 15 min at 4°C. CSF and plasma samples were aliquoted and frozen at -80°C until use.

#### ***Material collected from animals***

CSF and plasma samples were also collected from four 3 month-old PS1conditional knockout mice (*PS1* cKO) and five littermates in a C57BL6/129 hybrid background. In these cKO mice, PS1 expression has been selectively eliminated in glutamatergic neurons from the postnatal forebrain at P18, and at 2–3 months of age revealed no detectable brain PS1 [50]. CSF (4-6 μL) was obtained by cisternal puncture with a needle inserted in the suboccipital region through the atlanto-occipital membrane, with a single incision of the subarachnoid space as previously described [51]. Blood was withdrawn (0.2 mL) by cardiac puncture with heparinized needles and syringes. CSF and plasma samples were centrifuged at 1000 × g for 10 min at 4˚C and the supernatants stored at -80°C until further analysis.

#### ***Material collected from cells***

SH-SY5Y cells (800,000 cells/well) were grown in 6 well plates and transfected with a construct encoding full-length PS1 [52] (or with the expression plasmid pcDNA3 (Invitrogen), using Lipofectamine 2000^®^ (Invitrogen) according to the manufacturer’s instructions.

### Western blotting and immunoprecipitation

CSF samples (30-40 μL from human subjects; pooled volume of 15 μL from 3 mice), or plasma samples (0.5 μL) were denatured at 50°C for 15 min or alternatively at 98°C for 5 min for other analyses. For BACE1 analysis CSF samples were denatured always at 98°C for 5 min. Samples were resolved by sodium dodecyl sulfate-polyacrylamide gel electrophoresis (SDS-PAGE) under fully reducing conditions. For blue-native gel electrophoresis, samples were analyzed as previously described [53] and NativeMark™ Unstained Protein Standards (Life Techologies) were used for molecular weight markers. Alternatively, human plasma samples were first depleted of highly abundant proteins using immunoaffinity-based chromatography (Seppro^®^ IgY14 spin column kit, GenWay Biotech Inc) prior to gel electrophoresis (25 μg of protein after protein depletion). The separated proteins were transferred to nitrocellulose membranes (Schleicher and Schuell Bioscience GmbH) and probed with PS1 antibodies to N-terminal amino acids 1-65 (Calbiochem), amino acids 21-34 (from Thermo Scientific), amino acids 1-20 (antibody 98/1, [54]); amino acids 303-316 in the loop region of the C-terminal (Sigma), or to amino acids 301-317 in the loop region of human PS1(antibody 00/2; [54]). CSF proteins were also probed for PS2 using the polyclonal rabbit antibody “00/12,” raised to residues 307–336 of the human PS2 loop domain [55]. BACE1 antibody was from Cell Signaling, nicastrin antibody was from Millipore, APH-1, PEN-2 and albumin antibodies were from Sigma. Individual blots were used for the different antibodies, to eliminate antibody cross-reactivity. Blots were incubated with the corresponding secondary antibody conjugated to horseradish peroxidase and the signal was detected using ECL Plus detection reagent according to the manufacturer’s instructions (GE Healthcare) in a Luminescent Image Analyzer LAS-1000 Plus (FUJIFILM). A control CSF sample was used to normalize the immunoreactive signal. For semi-quantitative studies, the intensity of immunoreactive bands was measured by densitometry using Science Lab Image Gauge v 4.0 software provided by FUJIFILM.

Immunoprecipitations were performed at 4°C by incubating 150 μL of CSF overnight with the primary N-terminal PS1 antibody 98/1, or alternatively with an antibody from Thermo Scientific, previously coupled to protein A-Sepharose by Dimethyl pimelimidate dihydrochloride (Sigma-Aldrich Co). Precipitated proteins were washed with PBS and eluted with 0.1 M glycine buffer at pH 2.5. After pH neutralization, supernatants were denatured in Laemmli sample buffer at 50°C for 15 min and subjected to SDS-PAGE, and separated proteins were transferred to nitrocellulose membranes. Blots were incubated with N-terminal (Calbiochem) and C-terminal (00/2) PS1 antibodies.

### Sucrose gradients

PS1 complexes were analysed by ultracentrifugation at 250,000 × *g* in a continuous sucrose density gradient (5-20%) for 4 hr at 4°C in a Beckman TLS 55 rotor. CSF aliquots (50 μL) were carefully loaded onto the top of the gradient containing 2 mL of 0.15 M NaCl, 50 mM MgCl_2_ and 0.5% Brij 97, in 50 mM Tris-HCl (pH 7.4). After centrifugation ~14 fractions were gently collected from the top of the tubes. Enzyme markers of known sedimentation coefficient, β-galactosidase, catalase and alkaline phosphatase were used in the gradients to determine the approximate sedimentation coefficients.

### Measurement of γ-secretase-mediated peptide cleavage

γ-Secretase activity was assayed as reported elsewhere [17] using an intramolecularly quenched fluorogenic peptide probe, in the absence or presence of the γ-secretase inhibitor L-685,458 (10 μM). Membranes prepared from human brain solubilized in assay buffer containing 1% CHAPSO were used as positive controls [17]. Background fluorescence of the peptide probe was subtracted from all readings.

### Measurement of T-tau, P-tau and Aβ42by ELISA

Total tau (T-tau), phosphorylated tau (P-tau) and Aβ1-42 (Aβ42) in CSF were determined using specific enzyme-linked immunosorbent assays (ELISA) (Innogenetics, Ghent, Belgium).

### Statistical analysis

All data were analyzed in SigmaStat (Version 2.0; SPSS Inc.) by Student’s *t* test (two-tailed) or Mann-Whitney *U* test for single pair-wise comparisons and determination of exact *p* values. Results are presented as means ± SEM. Correlation between variables was assessed by linear regression analyses. *p* values < 0.05 were considered significant.

This study was approved by the ethics committee of the Miguel Hernandez University and was carried out in accordance with the Declaration of Helsinki.

## Abbreviations

Aβ: β-amyloid peptide; Aβ42: Aβ1-42; AD: Alzheimer’s disease; APH-1: anterior pharynx-defective 1; BACE1: beta-site APP cleaving enzyme 1; CTF: C-terminal fragment; CSF: Cerebrospinal fluid; NDC: Non-demented control; NTF: N-terminal fragment; PEN-2: Presenilin enhancer 2; PS1: Presenilin-1; PS2: Presenilin-2; P-tau: Hyperphosphorylated tau; SEM: Standard error of mean; T-tau: Total tau.

## Competing interests

All authors have contributed to the work and agree with the presented findings.

MSGA and JSV submitted a patent application for the use of PS1 complexes as an AD biomarker, as described herein. Appropriate procedures were followed to obtain approval from local ethics committees.

## Authors’ contributions

MSGA designed experiments, generated and analyzed data (WB, native electrophoresis, ultracentrifugation). MLC generated and analyzed data (WB, IP and γ-secretase activity assay). GB generated and analyzed data (IP and ELISA). AR provided brain and *post mortem* CSF samples. JA provided freshly collected *ante-mortem* CSF samples. CAS provided *PS1* cKO mice material and tested antibodies. NA provided CSF samples from AD and control subjects and supplied clinical data. KB designed experiments. JSV designed experiments, analyzed data and wrote the manuscript. All the authors participated in the preparation of the manuscript. All the authors contributed to the discussion of results and participated in the preparation of the manuscript. All authors read and approved the final manuscript.
